# STOP-BANG questionnaire as a screening tool for diagnosis of obstructive sleep apnea by unattended portable monitoring sleep study

**DOI:** 10.1186/s40064-015-1588-0

**Published:** 2015-12-22

**Authors:** Viral Doshi, Reuben Walia, Kellie Jones, Christopher E. Aston, Ahmed Awab

**Affiliations:** Division of Pulmonary, Critical Care and Sleep Medicine, University of Oklahoma Health Science Center and Oklahoma City VA Medical Center, Oklahoma City, OK USA; 920 Stanton L Young Blvd, WP 1310, Oklahoma City, OK 73104 USA; Department of Pediatrics, University of Oklahoma Health Sciences Center, Oklahoma City, OK 73104 USA

**Keywords:** Unattended portable monitoring, STOP-BANG, AHI, Home sleep study

## Abstract

The Snoring, Tiredness, Observed apnea, high blood Pressure (STOP)-Body mass index (BMI), Age, Neck circumference, and Gender (BANG) questionnaire is a well validated screening tool for diagnosis of Obstructive sleep apnea (OSA) by an in- lab sleep study. However, performance of STOP-BANG as a screening tool for diagnosis of OSA in patients undergoing portable monitoring (PM) sleep study has not been well validated. We conducted a retrospective chart review of patients older than 18 years who had unattended portable monitoring sleep study done at a VA medical center between June 2012 and October 2014. STOP-BANG questionnaire and Epworth sleepiness scale (ESS) were routinely done prior to study. Sensitivity, specificity, and positive predictive value (PPV) various STOP-BANG score thresholds were calculated for diagnosis of OSA defined by Apnea Hypopnea Index (AHI) ≥5. Out of 502 unattended portable monitoring sleep studies, there were 465 males and 37 females. STOP-BANG thresholds of ≥2 and 3 have high sensitivity of 99.8 and 98.9 %, respectively, but very low specificity. Higher score thresholds of ≥7 and 8 have high specificity of 95 and 98.3 %, and PPV of 98.1 and 98.5 %, respectively, but very low sensitivity. A threshold of ≥7 in patients with BMI ≥30 was 100 % specific. The false negative rate for unattended portable monitoring sleep study compared to in-lab study was 80 %. STOP-BANG score thresholds of ≥7 and 8 are highly specific and have high PPV and therefore can potentially reduce need of diagnostic sleep studies in selected patients. Score thresholds of ≤2 or 3 are highly sensitive for AHI ≥5 by unattended portable monitoring sleep study but have high false negative rates. Therefore, in-lab sleep study should be performed to rule out OSA.

## Background

Obstructive sleep apnea (OSA) has been shown to be independently associated with increased cardiovascular morbidity, including hypertension, congestive heart failure, ischemic heart disease, and stroke (Marin et al. 2005; Yaggi et al. 2005; Bradley and Floras 2009). Treatment of OSA can reduce daytime sleepiness and improves motor vehicle driving safety (Giles et al. 2006; Tregear et al. 2010). Polysomnography (PSG) is considered to be the gold standard for diagnosis of OSA. However, it is time-consuming and there is a long waiting period at many centers. Consequently, unattended portable monitoring (PM) has been increasingly used for the diagnosis of OSA (Collop et al. 2007).

The Snoring, Tiredness, Observed apnea, high blood Pressure (STOP)-Body mass index (BMI), Age, Neck circumference, and Gender (BANG) questionnaire is a validated screening tool for identifying OSA in surgical patients (Chung et al. 2008). The STOP-BANG questionnaire has been found to be equally effective in detecting OSA in community dwelling samples (Silva et al. 2011), in patients referred to a sleep center for formal PSG (Farney et al. 2011), and in patients in sleep clinics (Luo et al. 2014). For all of these studies standard PSG was used for diagnosis of OSA (Chung et al. 2008; Silva et al. 2011; Farney et al. 2011; Luo et al. 2014).

Use of STOP-BANG questionnaire for detection of OSA by unattended monitoring sleep study might not yield similar results, as the Apnea-Hypopnea index (AHI) obtained from both methods can differ. AHI obtained from unattended monitoring study can often be underestimated as AHI is calculated based on total recording time rather than total sleep time (Collop et al. 2007). PM can also have technical limitations resulting from displacement of sensors. In this study, we aim to evaluate STOP-BANG questionnaire as a screening tool for diagnosis of OSA by unattended portable monitoring sleep study by calculating sensitivity, specificity and positive predictive value for different STOP-BANG score thresholds.

## Methods

We conducted a retrospective chart review study of patients over 18 years of age at the Oklahoma City Veterans Affairs Medical Center (OKC VAMC) who had an unattended portable monitor-ing (PM) sleep study done between June 2012 and October 2014. The study was approved by the OKC VAMC institutional review board (IRB).

The OKC VAMC home sleep apnea program receives requests for sleep studies from VAMC health care providers. Requests consist of an electronically submitted consultation request with a brief description of the sleep problem and include electronically captured listings of medications and active medical problems. The Director of Sleep Medicine Clinic reviews all requests. Patients with significant comorbidities such as chronic obstructive pulmonary disease, congestive heart failure or neuromuscular disease were excluded from having an unattended portable monitor-ing sleep study and in-lab PSG was recommended for those patients. STOP-BANG questionnaire and Epworth sleepiness scale (ESS) were routinely documented electronically by ordering physicians before ordering a PM sleep study. Portable monitoring studies were done irrespective of their STOP-BANG and ESS.

The PM sleep studies were performed using either Alice PDX or Stardust systems which were type III portable equipment devices which recorded flow using nasal pressure cannula, effort using zRIP abdominal and thoracic belts, body position using position sensors, oxygenation using pulse oximetry and EKG using chest leads. Equipment was provided by sleep technicians in clinic and patients were instructed on its use. PM sleep study was done for one night on each patient. The equipment was mailed back to the clinic by the patient and data were downloaded by a sleep technician. All studies were scored automatically then followed by a manual review by a sleep physician for accuracy before generating a report.

Data obtained from the medical records were:

1. Patient demographics: age, sex and body mass intex (BMI)
2. STOP-BANG questionnaire score
3. Epworth sleepiness scale (ESS) score
4. Apnea-hypopnea index (AHI) by unattended portable monitoring (PM) study
5. AHI by polysomnography (if done).

### Inclusion and exclusion criteria

Inclusion criteria: any patient 18–99 years old with an unattended PM sleep study done at the OKC VAMC between June 2012 and October 2014. Exclusion criteria: patients with a non-diagnostic study due to technical reasons; patients with incomplete data.

### Statistical methods

Group data are expressed as mean ± SD or counts (%). The different groups will be compared using the unpaired t test and Fisher exact test. Significance will be accepted when the two-tailed p value is < 0.05. We evaluated the performance of various cut points of the STOP-BANG score by estimating the sensitivity and specificity for the presence of sleep apnea defined as AHI ≥5 by unattended PM sleep study. The area under the entire receiver operating characteristic curve (ROC AUC) is reported as a measure of overall discrimination. PPVs were calculated as the percentage of patients with a particular STOP-BANG score or higher who had a positive diagnostic sleep study that is AHI ≥5. Statistical analyses were conducted with SPSS software.

## Results

A total of 502 patients with unattended PM sleep studies were included in this study; averaging 50 years old, most were male (92 %) and obese (70 % with a BMI of 30 or higher (Table 1). The limited number of females tended to be younger (average age 47 vs 50 years) but had higher BMI (average BMI 38 vs 33, p < 0.0001) with 97 % of females having a BMI of 30 of higher. Thirty eight studies were excluded from analysis due to technical failure and incomplete data.

**Table 1:** Demographics and sleep study measures

	Total	Male	Female
N (%)	502	465 (92 %)	37 (8 %)
Age (years)	50 ± 14	50 ± 14	47 ± 12
BMI (kg/m^2^)	33 ± 6	33 ± 6	38 ± 6
N with BMI ≥ 30 (%)	353 (70 %)	317 (68 %)	36 (97 %)
AHI by unattended portable monitoring study	21 ± 18	22 ± 18	11 ± 13
STOP BANG questionnaire score	5.7 ± 1.5	5.7 ± 1.5	4.7 ± 1.2
Epworth sleepiness scale	11.7 ± 5.2	11.6 ± 5.2	13.0 ± 5.6

**Fig. 1:**
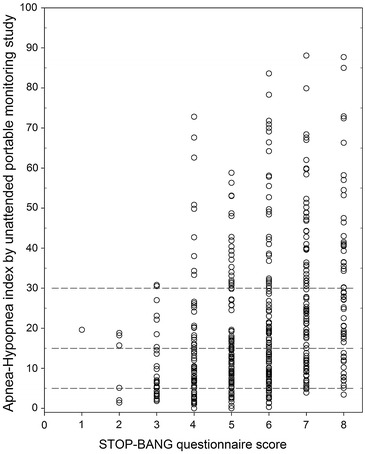
Distribution of mean AHI by STOP-BANG scores

The STOP-BANG scores in this sample ranged from 0 to 8, so all possible STOP-BANG scores were observed. The average AHI by PM increased as STOP-BANG score increased. The average STOP-BANG score of study population (Table 1) was 5.7 and was significantly lower in females compared to males (4.7 vs 5.7, p < 0.0001). The average AHI by PM score of the study was 21 (range: 0–88) and was significantly lower in females compared to males (11 vs 22, p < 0.0001). Figure 1 shows the distribution of AHI by PM for each STOP-BANG score in our study population. Average AHI by PM increased as the STOP-BANG score increased.

**Fig. 2:**
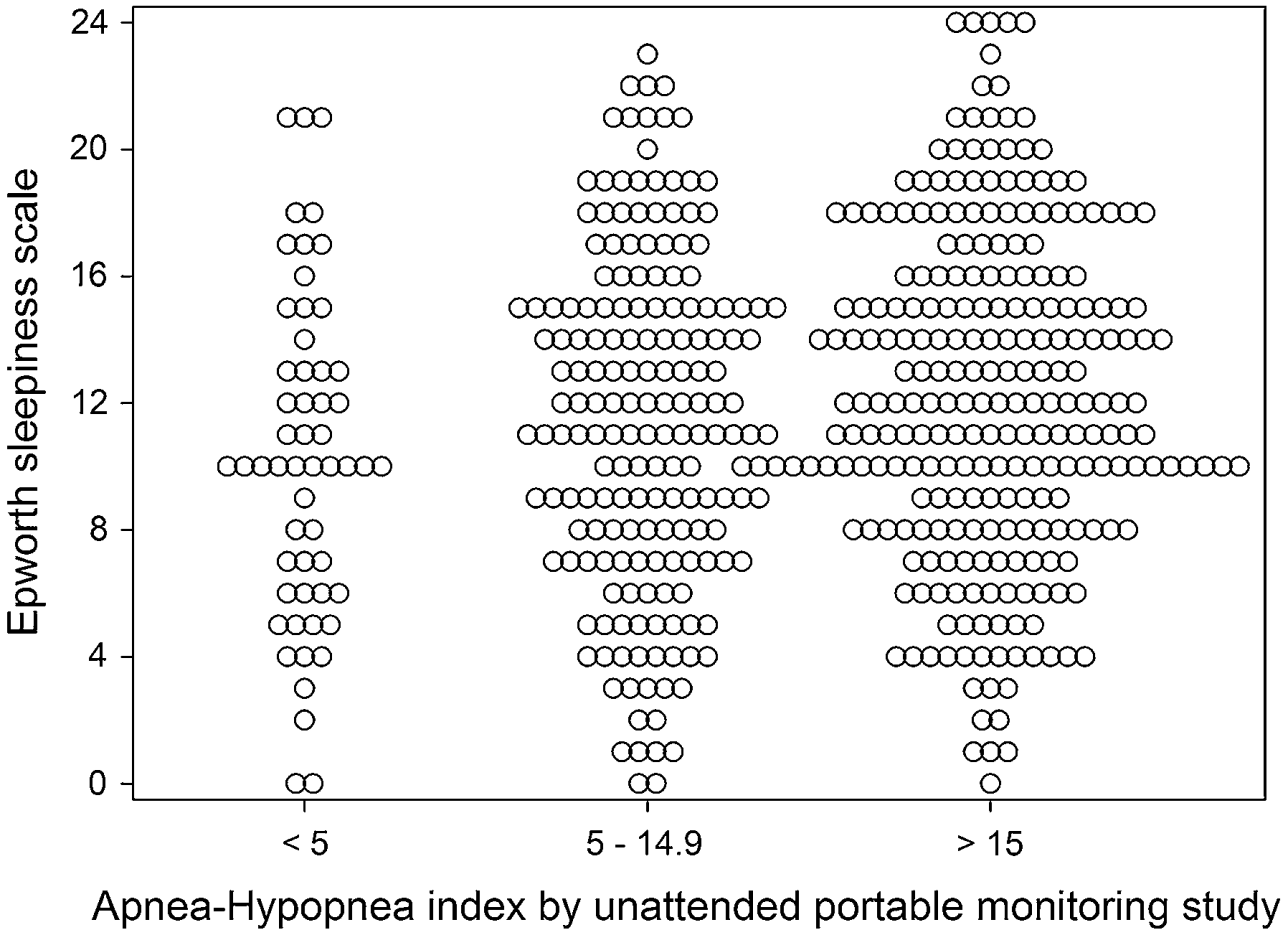
Distribution of mean ESS by STOP-BANG scores

The Epworth sleepiness scale (ESS) consisting of eight questions each scored 0–3 so the total score ranges from 0 to 24 and the full range was seen in our study. The average ESS of the study population was 11.7 and was not significantly different between males and females (Table 1). Figure 2 shows the distribution of ESS scores for AHI by PM ≤ 5, 5–14.9 and ≥15. There does not appear to be a relationship between ESS and AHI by PM scores.

Sensitivities and specificities for STOP-BANG scores to predicting an AHI by PM score ≥5/h are shown in Table 2. Sensitivity was excellent for lower STOP-BANG score thresholds, but sharply decreased for thresholds of 5 and above (Table 2). Specificity was high for STOP-BANG score thresholds of 7 and above. As a predictor of an AHI by PM score ≥5/h the ROC AUC for STOP-BANG scores was 0.72 (95 % confidence interval: 0.69–0.78). STOP-BANG scores were a significant predictor of AHI by PM score ≥5/h (p < 0.0001).

**Table 2:** Sensitivity, specificity and positive predictive value for various STOP-BANG thresholds to predict an apnea-hypopnea index (AHI) ≥5 by unattended portable monitoring sleep study

STOP-BANG score ≥	Sensitivity (%)	Specificity (%)	Predictive value	
			Positive (%)	Negative (%)
1	100.0	0.0	88.0	–
2	99.8	0.0	88.0	0.0
3	98.9	3.3	88.3	28.6
4	94.1	21.7	89.8	33.3
5	80.3	45.0	91.5	23.7
6	58.4	73.3	94.2	19.3
7	35.3	95.0	98.1	16.6
8	14.7	98.3	98.5	13.5

Sensitivity and specificity were calculated separately for males and females but yielded similar results for each (Table 3). We also calculated sensitivity and specificity for BMI <30 and ≥30 (Table 4). A STOP-BANG score threshold of ≥7 in patients with BMI ≥30 was 100 % specific for AHI by PM ≥5/h. Positive predictive value (PPV) exceeded 98 % for STOP-BANG score thresholds of ≥7 and 8. Thus, using a STOP-BANG score of 7 or higher would possibly obviate need of PM sleep study.

**Table 3:** Sensitivity, specificity and positive predictive value for various STOP-BANG thresholds to predict an apnea-hypopnea index (AHI) ≥ by unattended portable monitoring sleep study for male and female

STOP-BANG score ≥	Sensitivity		Specificity	
	Male (n = 465)	Female (n = 37)	Male (n = 465)	Female (n = 37)
1	100.0	100.0	0.0	0.0
2	99.8	100.0	0.0	0.0
3	98.8	100.0	4.0	0.0
4	95.2	77.8	24.0	10.0
5	82.4	48.1	50.0	20.0
6	60.0	33.3	68.0	100.0
7	37.1	7.4	94.0	100.0
8	15.7	0.0	98.0	100.0

**Table 4:** Sensitivity, specificity and positive predictive value for various STOP-BANG thresholds to predict an apnea-hypopnea index (AHI) ≥5 by unattended portable monitoring sleep study for BMI ≥30 and BMI < 30

STOP-BANG score ≥	Sensitivity		Specificity	
	BMI < 30 (n = 149)	BMI ≥ 30 (n = 353)	BMI < 30 (n = 149)	BMI ≥ 30 (n =353)
1	100.0	100.0	0.0	0.0
2	100.0	99.2	0.0	0.0
3	100.0	95.8	0.0	0.0
4	97.8	84.2	16.1	10.0
5	87.6	60.8	35.5	20.0
6	69.3	29.2	67.7	100.0
7	44.1	11.7	96.8	100.0
8	18.6	4.2	100.0	100.0

In lab polysomnography was done for ten patients that were negative (AHI < 5) in the unattended PM sleep studies. Of these ten, only two had AHI <5 in the PSG studies giving a very high false negative rate of 80 ± 12 %. For patients with AHI scores by both PM and PSG the average AHI by PM was 5.1 compared to 11.4 for AHI by PSG showing that PM underestimates AHI if AHI by PSG is taken as the gold-standard.

## Discussion

The standard of care at the OKC VAMC home sleep apnea program begins with request for sleep studies from VAMC health care providers. After review, and if appropriate, an unattended PM sleep study is performed to obtain an AHI by PM. If this score is 15 or 5 and greater and patient is symptomatic or has comorbidities then OSA is diagnosed and the patient is moved on to treatment; if it is less than 5 then an in lab polysomnography is performed. PM is relatively inexpensive but there can be inconclusive study due to technical reasons. Our results suggest that using a STOP-BANG questionnaire with a score threshold of ≥7 would possibly obviate the need for the PM study and can potentially reduce medical costs.

With an area under the ROC curve of 0.72 the STOP-BANG score was a fair predictor of whether a patient would have an AHI by PM score ≥5/h. A STOP-BANG score threshold of ≥7 was had high specificity (95 %) and positive predictive value (98 %). A positive result in a highly specific test is used to rule in disease, meaning in this case that a positive result (a STOP-BANG score ≥7) predicts AHI by PM ≥5. Applying this STOP-BANG threshold of ≥7 to the data used in this study, 159 patients had a STOP-BANG score of 7 or higher. Of these, 156 had AHI by PM scores ≥5/h and were diagnosed as OSA and treated accordingly. This can be useful especially in veteran affairs medical center to reduce cost of few studies. However, it might not be practical to use outside VA system as insurance requires a sleep study before reimbursing for CPAP device for treatment.

STOP-BANG score thresholds of ≥2 and 3 had high sensitivities of 99.8 and 98.9 %, respectively. These increased to 100 % if it was noted that the patient was obese (BMI ≥ 30). A negative result in a highly sensitive test is used to rule out disease, meaning in this case that a negative result with these STOP-BANG thresholds predicts AHI by PM <5, that is, a negative study by PM. PM tends to underestimate AHI compared to PSG making PM sleep studies less sensitive than in-lab sleep study (Collop et al. 2007) and seen in our data. Therefore, for STOP-BANG scores ≤2 or 3 one may move directly to standard in lab polysomnography to rule out obstructive sleep apnea due to the high false positive rate of the PM sleep study. Although in clinical practice, unattended portable monitoring studies can still be used as a starting point as it is much cheaper compared to standard polysomnography and one can still reduce overall cost of healthcare by reducing few in lab polysomnography studies.

Another study done by Kunisaki et al. (2014) looked at STOP-BANG Questionnaire performance in a Veterans Affairs unattended sleep study program used peripheral arterial tonometry (PAT) to diagnose OSA while our study used type III portable equipment (Collop et al. 2011) that is more commonly used and has more studies are available validating its use in clinical practice (Collop et al. 2011). Kunisaki et al. (2014) did not find high specificity or PPV even at high STOP-BANG thresholds of ≥7 or 8. This could be due to their use of a different AHI cutoff of 15 or above to diagnose OSA. Many patients with AHI between 5 and 15/h are symptomatic and have comorbidities that warrant treatment of OSA (Weaver et al. 2012). In our study 110 (61 %) of 179 patients with an AHI by PM between 5 and 15 had excessive daytime sleepiness suggested by ESS scale of 10 or more. Therefore, the AHI cut-off of 5 or more used in our study is probably more appropriate. Furthermore, it is known that PM studies tend to underestimate AHI (as measured by PSG) giving another reason to use the lower cut off for AHI (Collop et al. 2007).

Our study has a number of limitations. It is a retrospective study and suffers the potential limitations inherent to retrospective studies such as selection bias. Most patients were males, obese and had high STOP-BANG scores which could have biased conclusions. Also we had very few females in our study so these findings may not be as applicable to females. We could not confirm accuracy of STOP-BANG scores entered by providers. We did not measure if higher STOP-BANG scores are associated with more severe forms of OSA as primary role of the STOP-BANG questionnaire was to identify a high risk population for sleep apnea rather than determining severity.

In conclusion, in our unattended sleep study program at VA medical center, we found that High STOP-BANG thresholds of ≥7 or 8 can potentially alleviate need for few diagnostic studies. However, Lower STOP-BANG thresholds are not useful to rule out disease and formal in-lab sleep study should be done to rule out OSA due to high false negative rates of unattended portable monitoring studies at lower STOP-BANG thresholds.
